# Proteomic biomarkers predicting lymph node involvement in serum of cervical cancer patients. Limitations of SELDI-TOF MS

**DOI:** 10.1186/1477-5956-10-41

**Published:** 2012-06-13

**Authors:** Toon Van Gorp, Isabelle Cadron, Anneleen Daemen, Bart De Moor, Etienne Waelkens, Ignace Vergote

**Affiliations:** 1 Department of Obstetrics and Gynaecology, Leuven Cancer Institute, Universitaire Ziekenhuizen Leuven, KU Leuven, Herestraat 49, 3000, Leuven, Belgium; 2 Department of Obstetrics and Gynaecology, MUMC+, GROW – School for Oncology and Developmental Biology, P. Debyelaan 25, 6229 HX, Maastricht, The Netherlands; 3 Department of Obstetrics and Gynaecology, AZ Turnhout, Steenweg op Merksplas 44, 2300, Turnhout, Belgium; 4 Department Laboratory Medicine, UCSF School of Medicine, Box 0808, 2340 Sutter Street, MZ, San Francisco, CA, 94143, USA; 5 Department of Electrical Engineering, ESAT-SCD/SISTA, Kasteelpark Arenberg 10, PO box 2446, 3001, Heverlee, Belgium; 6 Department of Molecular Cell Biology, Universitaire Ziekenhuizen Leuven, Katholieke Universiteit Leuven, Herestraat 49, 3000, Leuven, Belgium

**Keywords:** Cervical cancer, Biomarker, Recurrence, Lymph node, SELDI-TOF MS

## Abstract

**Background:**

Lymph node status is not part of the staging system for cervical cancer, but provides important information for prognosis and treatment. We investigated whether lymph node status can be predicted with proteomic profiling.

**Material & methods:**

Serum samples of 60 cervical cancer patients (FIGO I/II) were obtained before primary treatment. Samples were run through a HPLC depletion column, eliminating the 14 most abundant proteins ubiquitously present in serum. Unbound fractions were concentrated with spin filters. Fractions were spotted onto CM10 and IMAC30 surfaces and analyzed with surface-enhanced laser desorption time of flight (SELDI-TOF) mass spectrometry (MS). Unsupervised peak detection and peak clustering was performed using MASDA software. Leave-one-out (LOO) validation for weighted Least Squares Support Vector Machines (LSSVM) was used for prediction of lymph node involvement. Other outcomes were histological type, lymphvascular space involvement (LVSI) and recurrent disease.

**Results:**

LSSVM models were able to determine LN status with a LOO area under the receiver operating characteristics curve (AUC) of 0.95, based on peaks with m/z values 2,698.9, 3,953.2, and 15,254.8. Furthermore, we were able to predict LVSI (AUC 0.81), to predict recurrence (AUC 0.92), and to differentiate between squamous carcinomas and adenocarcinomas (AUC 0.88), between squamous and adenosquamous carcinomas (AUC 0.85), and between adenocarcinomas and adenosquamous carcinomas (AUC 0.94).

**Conclusions:**

Potential markers related with lymph node involvement were detected, and protein/peptide profiling support differentiation between various subtypes of cervical cancer. However, identification of the potential biomarkers was hampered by the technical limitations of SELDI-TOF MS.

## Background

Cervical cancer is the seventh most common cancer in both sexes combined and the third most common cancer in women. An estimated 530,000 women across the world were diagnosed with cervical cancer in 2008, accounting for nearly one in ten (9%) of all cancers diagnosed in women. The developing countries carry the biggest burden of cervical cancer, with more than 450,000 cases being diagnosed in 2008 [1].

Lymph node (LN) status is not part of the staging system of the International Federation of Gynecology and Obstetrics (FIGO) for cervical cancer [2], but it provides important information for prognosis and treatment, in particular in early stage cervical cancer [3,4]. The incidence of pelvic LN metastases varies from 0–2% in FIGO stage IA, 17–24% in FIGO stage IB1, 17–50% in FIGO stage IB2, and 10–50% in FIGO stage IIa [4-10].

In patients with early stage cervical cancer, the treatment of choice is either surgical, including radical hysterectomy and pelvic LN dissection, or chemoradiation. Combining both treatments leads to a higher morbidity, such as lymph edema and urological complications [11]. Specifically for patients with lymph node metastases, chemoradiation is the treatment of choice since it reduces local and distant recurrences [12]. Preoperative diagnostic modalities such as CT scan and MRI have a good specificity, but a low sensitivity [13,14]. This explains why a certain number of patients, in whom the diagnosis of positive LN is only made after pathological examination, still receive a combined treatment of surgery and pelvic irradiation.

Various proteomics techniques have been used to detect new biomarkers in gynaecological cancers with variable degrees of success [15]. Over the last decade, surface-enhanced laser desorption time of flight (SELDI-TOF) mass spectrometry (MS) has been a popular proteomics technique due to its ease of use and high throughput. Several studies have published comparative studies on new diagnostic proteins [15].

We investigated whether we could improve the prediction of LN involvement with SELDI-TOF MS proteomic profiling.

## Results

### Patients

Patient and tumour characteristics are represented in sTable 1. Twelve patients were diagnosed with positive LNs. The remainder of the patients had a complete lymphadenectomy performed, but no positive lymph nodes were diagnosed. Both groups were well balanced for age, FIGO stage, histological subtype, number of removed LNs, incidence of LVSI, duration of follow-up and incidence of recurrence. LVSI was—as expected—associated with LN status.

**Table 1:** Patient and tumour characteristics

	**Numerical display**	**LN positive (n = 12)**	**LN negative (n = 48)**	**Test**	**P value**
**Age in years**	Mean (95%CI)	45.8 (38.5–53.0)	46.7 (43.3–50.0)	*T*-test	0.732
**FIGO stage**					
Ia2	n (%)	0 (0.0)	2 (4.2)	*χ*^2^	0.134
Ib1	n (%)	6 (50.0)	37 (77.1)		
Ib2	n (%)	2 (16.7)	2 (4.2)		
IIa	n (%)	4 (33.3)	7 (14.6)		
**Histological subtype**					
Squamous cell carcinoma	n (%)	11 (91.7)	29 (60.4)	*χ*^2^	0.119
Adenocarcinoma	n (%)	1 (8.3)	17 (35.4)		
Adenosquamous carcinoma	n (%)	0 (0.0)	2 (4.2)		
**Lymph nodes**					
Number of positive LN	Median (min-max)	1 (1–7)	0 (0–0)	Mann–Whitney	<0.001
Number of removed LN	Median (min-max)	28 (4–50)	34 (18–89)	Mann–Whitney	0.241
**LVSI**					
Positive	n (%)	10 (83.3)	16 (33.3)	*χ*^2^	0.005
Negative	n (%)	2 (16.7)	32 (66.7)		
**Follow-up**					
Follow-up (in months)	Mean (95%CI)	61.8 (42.0–81.5)	61.7 (49.0–66.5)	*T*-test	0.997
**Recurrence**					
Recurrence	n (%)	3 (25.0)	7 (14.6)	*χ*^2^	0.665
No recurrence	n (%)	9 (75.0)	41 (85.4)		

Abbreviations: LN = lymph node; FIGO = International Federation of Gynecology and Obstetrics; LVSI = lymphvascular space involvement.

### Unsupervised peak detection

In total 597 different peaks were detected in our panel of 60 samples: 284 peaks on CM10 and 313 on IMAC30. In Table 2 the number of peaks that was differentially expressed according to LN status, histological subtype, LVSI and recurrence of disease are shown. In general, the number of differentially expressed peaks was higher in the low mass range, except for the difference between squamous carcinomas and adenocarcinomas. The total number of differentially expressed peaks ranged from 11 to 37, depending on the comparison which was made. A complete list of the m/z values of the differentially expressed peaks with corresponding p-values is provided in Additional file 1.

**Table 2:** The total number of identified peaks and the number of peaks that was significantly differentially expressed for the given comparisons

	**CM10**		**IMAC**		**Total**
	**Low mass <10 kDa**	**High mass >10 kDa**	**Low mass <10 kDa**	**High mass >10 kDa**	
**Total number of peaks**	175	109	172	141	**597**
**Lymph node status**					
Negative *vs* Positive	2	0	5	5	**12**
**Histological subtype**					
Squamous ca. *vs* Adenoca.	8	15	3	5	**31**
Squamous ca. *vs* Adenosquamous ca.	4	2	4	1	**11**
Adenoca. *vs* Adenosquamous ca.	3	0	18	0	**21**
**LVSI**					
Negative *vs* Positive	18	0	14	5	**37**
**Recurrence**					
Negative *vs* Positive	4	0	7	3	**14**

Abbreviations: CM10 = weak cation exchanger array; IMAC = immobilized metal affinity capture array; ca. = carcinoma; LVSI = lymph vascular space involvement.

### LOO internal validation for weighted LSSVM

**Figure 1:**
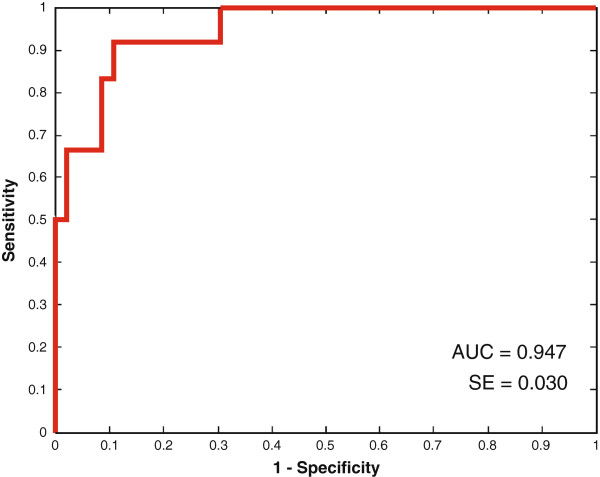
**Receiver operating characteristics (ROC) curve for the prediction of lymph node status.** Abbreviations: AUC: area under the curve, SE = standard error.

The AUC values obtained by LOO internal validation with the optimal median and mean number of peaks across all LOO iterations are represented in Table 3. For the prediction of LN status an AUC value of 0.95 was obtained (Figure 1). Three peaks were repeatedly selected in the LOO iterations: m/z values 2,698.9, 3,953.2, and 15,254.8 from the IMAC low mass, CM10 low mass, and IMAC high mass spectra, respectively (Table 4).

**Table 3:** AUC obtained by leave-one-out internal validation (LOO) with the optimal median and mean number of peaks per iteration

	**LOO AUC (SE)**	**Sensitivity**	**Specificity**	**Median number of peaks per LOO iteration**	**Mean number of peaks per LOO iteration (SD)**
**Lymph node status**					
Negative *vs* Positive	0.95 (0.03)	73.9	91.7	1	1 (0)
**Histological subtype**					
Squamous ca. *vs* adenoca.	0.88 (0.05)	88.2	59.0	1	1 (0)
Squamous ca. *vs* adsq ca.	0.85 (0.06)	84.6	100	4	3.8 (0.9)
Adenoca. *vs* adsq ca.	0.94 (0.06)	94.1	100	1	0.9 (0.3)
**LVSI**					
Negative *vs* Positive	0.81 (0.06)	78.1	73.1	1	1 (0)
**Recurrence**					
Negative *vs* Positive	0.92 (0.04)	79.2	90.0	3	3 (0)

Abbreviations: SE = standard error; SD = standard deviation; ca. = carcinoma; Adsq ca. = Adenosquamous carcinoma; LVSI = lymph vascular space involvement.

**Table 4:** Most frequent selected peaks in the leave-one-out internal validation (LOO) iterations, with the corresponding chip surface and mass range

	**Median m/z value**	Occurrence*	**p-value**	**Chip surface**	**Mass range**^**§**^
**Lymph node status**					
Negative *vs* Positive					
	2698.945	15	0.023	IMAC	Low
	15254.808	14	0.022	IMAC	High
	3953.177	13	0.024	CM10	Low
**Histological subtype**					
Squamous ca. *vs* Adenoca.					
	12802.775	28	0.021	CM10	High
	78632.414	11	0.020	IMAC	High
Squamous ca. *vs* Adenosquamous ca.					
	1532.112	39	0.032	CM10	Low
	1532.166	39	0.032	CM10	Low
	1627.269	39	0.032	IMAC	Low
	4783.483	38	0.032	IMAC	Low
Adenoca. *vs* Adenosquamous ca.					
	1531.463	16	0.012	CM10	Low
**LVSI**					
Negative *vs* Positive					
	1741.204	58	0.008	IMAC	Low
	3224.349	13	0.010	CM10	Low
**Recurrence**					
Negative *vs* Positive					
	94029.326	53	0.021	IMAC	High
	97177.269	52	0.018	IMAC	High
	78294.986	52	0.027	IMAC	High
	2044.703	21	0.031	IMAC	Low
	1979.514	15	0.035	IMAC	Low

* The number of times the peak was selected within the different LOO iterations.

§ Low mass range: <10 kDa; high mass range >10 kDa.

Abbreviations: CM10 = weak cation exchanger array; IMAC = immobilized metal affinity capture array; ca. = carcinoma; LVSI = lymph vascular space involvement.

LVSI was more difficult to predict. Although a median number of one peak was sufficient, the LOO AUC reached only a value of 0.81. A median number of 1 peak was needed to construct a model that was able to differentiate squamous carcinomas with adenocarcinomas (AUC 0.88), 4 peaks to differentiate between squamous and adenosquamous carcinomas (AUC 0.85), 1 peak to differentiate between adenocarcinomas and adenosquamous carcinomas (AUC 0.94), and 3 peaks to predict recurrence (AUC 0.92). The most frequently selected peaks for the different comparisons are represented in Table 4.

## Discussion

This study investigated whether we could improve the prediction of LN involvement with proteomic profiling. We used a combination of HPLC immunodepletion with SELDI-TOF MS to detect proteins that predict LN involvement. Using LSSVM models we were able to predict lymph node involvement with an AUC of 0.95. These findings suggest that serum biomarkers could help us identifying patients with LN metastases. Other outcomes, such as histological type (AUC = 0.85–0.94), lymph vascular space involvement (AUC = 0.81) and recurrence (AUC = 0.92), were also successful, however the number of patients in some of the subgroups was limited (e.g. adenosquamous subtype (n = 2)) making the results less reliable.

The majority of serum proteins are high-abundance proteins, accounting for almost 99% of the total protein mass [16]. Most of these proteins are true serum or plasma proteins that carry out their functions in the circulation, rather than proteins or peptides that leak into the blood (e.g. tumor tissue proteins) [16]. Removing the high abundant proteins facilitates the discovery and identification of low-abundance proteins that may be biomarkers [17]. The MARS-14 immunodepletion column used in the present study removes 95–99% of the 14 most abundant proteins from serum, thereby increasing the likeliness of finding possible biomarkers [18,19]. This technique has proven to be highly reproducible [19]. However, due to protein–protein or protein–antibody interactions also non-targeted proteins are being removed [19,20] which could hamper the detection of certain proteins. Moreover, some reports mention that the detection of medium abundance proteins improves, but not the detection of the very low abundance proteins (<10 ng/mL) [18]. This is the range in which some of the currently known biomarkers are found (e.g. CEA) [16]. Another problem with immunodepletion in combination with SELDI-TOF MS is that both systems, the HPLC and SELDI-TOF MS are not in-line as other LC-MS techniques. The additional sample handling introduces additional experimental variables, such as additional freezing/thawing cycles, and manually handling of the samples.

Upon establishing the biomarker profiles for lymph node involvement in cervical cancers, it became interesting to identify the proteins behind the differentially expressed peaks. For the 15,254.8 peak detected on the IMAC30 chip, an approach was developed using immunodepletion and SDS-PAGE gel electrophoresis as initial separation steps. Unfortunately, due to the apparently very low concentration of this protein in serum, no Coomassie Blue band could be detected at the level of 15–16 kDa. For the two lower masses (2,698.9 and 3,953.2) an attempt was undertaken for direct identification from the corresponding SELDI target plate. This involved the use of a special SELDI Chip target adapter (Bruker Daltonics, Bremen, Germany) to analyze the spots with a matrix-assisted laser desorption/ionization (MALDI)-TOF/TOF MS (Ultraflex 2, Bruker Daltonics, Bremen, Germany). Indeed, the TOF/TOF MS can induce fragmentation of selected masses, which is essential for their subsequent identification. However, SELDI-TOF MS is known for having a poor mass accuracy or reproducibility [21]. This made it difficult to determine which peak in the 2,650–2,750 and the 3,900–4,000 Da range on MALDI-TOF MS/MS was responsible for the 2,698.9 and 3,953.2 peaks on SELDI-TOF. Moreover, collision induced dissociation (CID) of high mass peaks (>3 kDa) is difficult in currently available MALDI TOF/TOF MS instruments, yielding no or incomplete fragments from this masses. Alternatively, an off-line sample preparation was explored to allow analysis of larger volumes of samples using a MALDI target plate. In this project, SELDI-TOF MS on-chip chromatographic surfaces are used to select proteins with either cationic or metal affinity properties. This gives two advantages to SELDI-TOF MS: (1) the chromatographic surface acts as an additional fractionation step, selecting only a subset of proteins that will be analyzed (enrichment), and (2) the proteins get separated from salts and other sample contaminants by subsequent on-spot washing with appropriate buffer solutions. As in MALDI MS analysis, on-chip purification is not possible, sample cleanup procedures must be applied before the sample is put on the target to reduce noise and ion suppression. In our identification experiments we applied an additional desalting step by using revered phase chromatography, either by HPLC, or by C4 or C18 Zip-Tip. These additional steps introduced additional experimental variables making it even more uncertain to identify the correct protein. Taken together, the additional sample preparations resulted in sample loss as well as introducing qualitative and quantitative variances, without leading to the required identification.

When looking at the literature on SELDI-TOF experiments, it can be noticed that in only a minority of papers an identification was performed. Most of the papers mention that identification and validation of the newly discovered biomarkers is ongoing. However, follow-up papers on the identified proteins, or validation studies are rarely published. For example, SELDI-TOF MS was used to differentiate cervical cancer and normal cervix tissue in the study by Wong *et al.*[22]. The authors were able to discover a discriminatory peak profile with a sensitivity of 87% and a specificity of 100%. To the best of our knowledge there was no follow-up study published in which these results were validated or the proteins identified. Another example is the study by Lin *et al.*[23] in which plasma proteomic profiling with SELDI-TOF MS was used to differentiate in situ carcinoma and invasive carcinoma of the cervix. Although a very high sensitivity and specificity was found with a limited amount of differentially expressed peaks, there were no follow-up studies published. Furthermore, this is not only the case for biomarker discovery studies for gynecological cancers [15], but also for various other types of cancer [24,25]. This questions the utility/advantage of the using a SELDI-TOF MS approach. Over the last decade the field of mass spectrometry has evolved and expanded with new techniques: high-definition MS equipment and new software enables scientists to detect proteins up to the femtogram level. Future developments include tandem expansions with multiple connections to HPLC equipment. In-depth analyses of fluid or tissue specimens seems now possible. There is a place for a global proteomics approach, but this should be an in-depth proteomic profiling with high levels of fractionation, separation and identification.

## Conclusions

In conclusion, the SELDI TOF MS approach has allowed to discover a set of proteomic profiles (revealing potential biomarkers) that could help us in the diagnosis of LN metastases. However, the proteins/peptides concerned were not identified due to technical limitations of the SELDI-TOF MS technique.

## Material and methods

### Patients

Serum samples of 60 cervical cancer patients were obtained before primary surgery. All patients were diagnosed with FIGO stage I or II cervical cancer. Prior to enrolment in the study, all patients were required to give fully informed consent. The protocol was approved by the Local Ethics Committee (reference: 3M040097/ML2524).

### Depletion

For each of the 60 serum samples, immunodepletion was performed using a high capacity 4.6 × 100 mm multiple affinity removal system (MARS) column (Agilent Technologies, Diegem, Belgium) in an Agilent 1200 high pressure liquid chromatography (HPLC) system (Agilent Technologies, Diegem, Belgium). This column eliminates the 14 most abundant proteins ubiquitously present in serum: albumin, alpha1-acid glycoprotein, alpha2-macroglobulin, antitrypsin, apolipoprotein AI, apolipoprotein AII, complement C3, fibrinogen, haptoglobin, IgA, IgG, IgM, transferrin, and transthyretin. In brief, the serum samples were diluted four-fold with Buffer A (Agilent Technologies, Diegem, Belgium), filtered through a 0.22 mm spin filter and 100 μl of the diluted serum was injected into the column in 100% Buffer A at a flow rate of 0.125 mL/min. After collection of the flow-through (i.e. depleted fraction) for 5.5 min, the column was washed and the bound (high abundance) proteins were eluted with 100% Buffer B (Agilent Technologies, Diegem, Belgium) at a flow rate of 1 mL/min for 2.5 min. The column was re-equilibrated using 100% Buffer A. Protein elution was monitored at a wavelength of 280 nm during the chromatography fractionation process. Reproducibility and efficiency of MARS column was checked by inspecting the peak position and height of the flow trough and eluted proteins as well as the overlay of the first and last chromatogram of every column using pooled serum samples as controls.

### Concentration and buffer exchange

The collected flow-through fraction containing the low-abundant proteins was filtered using a 1,000 Da molecular weight Microsep spin filter (Pall, Zaventem, Belgium) for the low molecular weight analysis and a 5,000 Da molecular weight Agilent spin filter (Agilent, Diegem, Belgium) for the high molecular weight analysis. After a first filtration step at 7500 × g for 100 and 30 min for the 1,000 and 5,000 Da spin filter, respectively, a fixed amount of the SELDI-TOF MS binding buffer (CM10 and IMAC binding buffers: see below for specifications) was added and the filtration step was repeated. This last step (adding buffer + filtration) was repeated three times to perform a buffer exchange from Buffer A to the SELDI-TOF MS binding buffers. The samples were then stored at −80°C until further use.

### Protein profiling with SELDI-TOF MS

Fractions were analysed in duplicate on CM10 (weak cation exchanger) and copper-coated IMAC30 (immobilized metal affinity capture) arrays (Bio-Rad, Nazareth, Belgium). All samples were randomly assigned to the different spots. For the CM10 arrays, spots were pre-incubated twice with CM10 binding buffer (0.1 M sodium acetate, pH 4.0) followed by application of 100 μl of the sample in the same binding buffer. For the IMAC30 arrays, spots were pre-incubated twice with 50 μl of 0.1 M copper sulphate for 5 min at room temperature followed by a wash step with 0.1 M sodium acetate buffer pH 4 for 5 min at room temperature. Spots were then pre-incubated twice with IMAC30 binding buffer (0.1 M sodium phosphate, 0.5 M NaCl pH 7) followed by application of 100 μl of the sample in the same binding buffer. Samples were incubated for 60 min at 4°C with shaking on a MicroMix (Siemens Medical Solutions Diagnostics, Brussels, Belgium). After three additional wash steps with the same binding buffer and two final washes with water, 2 × 1 μl of 20% α-cyano-4-hydroxy cinnamic acid (CHCA) or 100% sinapinic acid (SPA) (Bio-Rad, Nazareth, Belgium) dissolved in 1% TFA/100% ACN were applied. CHCA was predominantly used to improve ionization for lower mass peaks (<10,000 Da) and SPA for the high mass peaks (10,000–100,000 Da). Mass analysis was performed using SELDI-TOF MS (PCS 4,000 Enterprise, Ciphergen ProteinChip Reader Inc., Fremont, CA) applying automated data collection protocols for a molecular weight of <10,000 Da (low molecular weight protocol) and for 10,000–100,000 Da (high molecular weight protocol). The following settings were used: (a) sampling rate 400 MHz; (b) 2 warming shots (not included in analysis), 10 data shots per point and (c) total number of points evaluated equal to 12.5% of the spot surface. The low and high molecular weight protocols were further optimized in pilot studies (data not shown) to reach an optimal number of peaks and signal to noise (S/N) ratio (the maximum number of peaks at S/N > 2 and S/N > 5 were counted per laser intensity). For the low molecular weight protocol a laser intensity of 2,500 nJ; focus mass 5,000 Da; and matrix attenuation 500 Da was chosen. For the low molecular weight protocol a laser intensity of 2,500 nJ; focus mass 19,000 Da; and matrix attenuation 5,000 Da was chosen. Mass accuracy was calibrated externally using the all-in-one peptide and all-in-one protein standard according to the manufacturer’s instructions (Bio-Rad) for the low and high molecular weight analysis, respectively. A quality control sample (pooled serum) was analyzed weekly to validate the output of the system. Pooled serum samples were also used as positive controls (one spot on every chip was randomly assigned) and run with the same protocol as the weekly control samples. Data analysis of the control samples was performed with Shewhart control charts plots [26]. The fulfillment of the following Westgard rules was checked: 1:3 s, 2:2 s, 4:1 s, 10×. The analysis of the quality control samples was within limits during the timeframe this study. Using the Ciphergen Express Software, baseline subtraction and noise reduction were completed before peak intensities were normalized to the total ion current of the experimental samples. Outlier spectra were identified and removed from the analyses when the normalisation factor deviated more than 2 standard deviations. Numeric data were exported to csv-files for further biostatistical processing.

### Data analysis

With the aid of MASDA software the following additional preprocessing steps were performed [27,28]: (1) peak detection based on changes in the first derivative of a sample’s intensity curve, (2) peak filtering with exclusion of peaks below a local noise threshold defined as the median plus five times the median absolute deviation, and (3) peak matching/alignment across samples using complete linkage hierarchical one-dimensional clustering. The significance of peaks was determined with the non-parametric Wilcoxon rank sum test. A p-value of <0.05 was deemed significant.

Weighted Least Squares Support Vector Machine (LSSVM) in combination with leave-one-out (LOO) cross-validation was used to build classifiers [29,30]. For the optimization of number of peaks included in the classifiers, the number of peaks tested within each LOO iteration ranged from 1 to maximum 10, only including significant peaks (p < 0.05). For both CM10 and IMAC30, the low mass and high mass peaks were simultaneously included in the models in order of decreasing significance. The optimal model parameter (regularization parameter of the weighted LSSVM) was chosen as the one corresponding to the largest area under the curve (AUC) of the receiver operating characteristic curve. When multiple parameters with the same AUC were present, the balanced error rate was minimized with an as high as possible sum of sensitivity and specificity. The main outcome was LN status (negative *vs* positive). Secondary outcomes were histological subtype, lymph-vascular space involvement (LVSI) and recurrent disease.

## Abbreviations

LN: Lymph node; FIGO: International Federation of Gynecology and Obstetrics; SELDI: Surface-enhanced laser desorption; TOF: Time of flight; MS: Mass spectrometry; MARS: Multiple affinity removal system; HPLC: High pressure liquid chromatography; CM10: Weak cation exchanger array; IMAC30: Immobilized metal affinity capture array; CHCA: α-cyano-4-hydroxy cinnamic acid; SPA: Sinapinic acid; S/N: Signal to noise ratio; LSSVM: Least Squares Support Vector Machine; LOO: Leave-one-out; AUC: Area under the curve; LVSI: Lymph-vascular space involvement; MALDI: Matrix-assisted laser desorption/ionization; CID: Collision induced dissociation; ca.: Carcinoma; SD: Standard deviation.

## Competing interests

The authors declare that they have no competing interests.

## Authors’ contributions

TVG, EW, and IV were responsible for planning and designing the study. TVG and IC collected samples. TVG and IC developed the protocols. TVG and EW performed the experiments. AD en BDM performed the statistical analysis. TVG wrote the manuscript. All authors read and approved the final manuscript.

## Financial disclosures

This work was supported, in part, by the Belgian Federation against Cancer, a non-profit organization (SCIE2004-42), and the Research Foundation – Flanders (FWO) (G.0457.05).

## Supplementary Material

Additional file 1 A complete list of differentially expressed peaks with corresponding m/z and p-values. Click here for file
