# Supplementary material for: Proteomic biomarkers predicting lymph node involvement in serum of cervical cancer patients. Limitations of SELDI-TOF MS
Source: Proteome Sci. 2012 Jun 13;10:41. doi: 10.1186/1477-5956-10-41 (PMC3493309; doi:10.1186/1477-5956-10-41)
Supplement: Additional file 1 — A complete list of differentially expressed peaks with corresponding m/z and p-values. [file 1477-5956-10-41-S1.pdf]

Additional file 1

Lymph node status: Negative *vs* Positive (n = 12)

Chip	Mass range	median MZ	P value
CM10	Low mass (< 10 kDa)	3953.1768	0.016701
		1528.0044	0.041142
IMAC	Low mass (< 10 kDa)	2698.9448	0.022927
		3155.546	0.022927
		3912.7152	0.029356
		4342.095	0.030814
		5395.1557	0.042857
	High mass (> 10 kDa)	15143.394	0.032802
		15234.882	0.039343
		15254.8075	0.039343
		95110.1205	0.039343
		16580.7285	0.04696

Histological type: Squamous carcinoma *vs* Adenocarcinoma (n = 31)

Chip	Mass range	median MZ	P value
CM10	Low mass (< 10 kDa)	3322.4354	0.029514
		4471.9029	0.034917
		3305.1314	0.036393
		3314.7043	0.036393
		6624.3513	0.036393
		6665.0278	0.039504
		1980.9877	0.046408
		2468.737	0.046408
	High mass (> 10 kDa)	12802.7745	0.020833
		12604.3585	0.021779
		12812.301	0.021779
		12842.759	0.021779
		12940.3205	0.030793
		17068.5485	0.031447
		25591.8345	0.032118
		12866.8525	0.033493
		14197.144	0.036393
		25615.244	0.039501
		12907.661	0.039504
		51184.452	0.044593
		51198.731	0.046408
		14133.569	0.048284
		14157.042	0.048284
IMAC	Low mass (< 10 kDa)	4181.0983	0.022554
		4673.3516	0.040438
		3504.9496	0.045957
	High mass (> 10 kDa)	78632.414	0.019922
		78776.973	0.022763
		78294.9855	0.027094
		78417.606	0.032118
		95794.578	0.044593

Histological type: Squamous carcinoma *vs* Adenosquamous carcinoma (n = 11)

Chip	Mass range	median MZ	P value
CM10	Low mass (< 10 kDa)	1532.1116	0.036019
		1532.1661	0.036019
		1518.8278	0.047862
		1531.4628	0.047862
	High mass (> 10 kDa)	95532.8225	0.047862
		95686.3475	0.047862
IMAC	Low mass (< 10 kDa)	1627.269	0.031669
		4783.4832	0.031669
		4126.0393	0.049184
		4181.0983	0.049184
	High mass (> 10 kDa)	91495.2565	0.047862

Histological type: Adenocarcinoma *vs* Adenosquamous carcinoma (n = 21)

Chip	Mass range	median MZ	P value
CM10	Low mass (< 10 kDa)	1531.4628	0.037635
		1532.1116	0.037635
		1532.1661	0.037635
IMAC	Low mass (< 10 kDa)	2184.8034	0.011696
		3771.2867	0.011696
		3970.1544	0.011696
		4435.1527	0.011696
		1532.2435	0.023392
		1554.3088	0.023392
		2427.6168	0.023392
		3065.4704	0.023392
		4126.0393	0.023392
		1517.5746	0.046784
		1518.8165	0.046784
		1545.0487	0.046784
		1594.4996	0.046784
		1786.169	0.046784
		2310.7389	0.046784
		3990.3588	0.046784
		4033.1544	0.046784
		4298.8249	0.046784

Lymphvascular space involvement: Negative *vs* Positive (n = 37)

Chip	Mass range	median MZ	P value
CM10	Low mass (< 10 kDa)	3324.0214	0.007089
		3224.3486	0.00775
		3338.1309	0.012465
		3331.0118	0.014727
		6439.0795	0.014727
		3322.4354	0.017343
		4641.1334	0.018797
		6665.0278	0.02291
		3305.1314	0.027782
		6420.1398	0.029968
		3239.7341	0.036089
		6551.6233	0.036089
		3222.5037	0.03882
		5061.0332	0.041725
		3969.9798	0.043245
		5317.6835	0.044811
		3293.5136	0.048089
		5332.5344	0.049801
IMAC	Low mass (< 10 kDa)	1741.2045	0.008045
		4245.1475	0.012092
		1754.2504	0.018618
		3239.0923	0.020242
		4359.1972	0.021989
		2861.0547	0.023866
		3259.4918	0.02588
		4033.1544	0.026941
		3261.2101	0.030351
		3223.0784	0.036861
		1803.3559	0.038297
		3792.7662	0.041311
		3236.1447	0.044524
		5244.2226	0.049737
	High mass (> 10 kDa)	39326.646	0.019564
		39419.8245	0.02203
		21993.502	0.036089
		29485.6895	0.048085
		29550.68	0.048089

Recurrence: Negative *vs* Positive (n = 14)

Chip	Mass range	median MZ	P value
CM10	Low mass (< 10 kDa)	7914.1556	0.032982
		7916.948	0.036382
		3872.7463	0.038191
		3881.2838	0.048425
IMAC	Low mass (< 10 kDa)	2044.703	0.031468
		1979.5139	0.034864
		2037.1915	0.038569
		2145.8063	0.040543
		3272.0415	0.040543
		3236.1447	0.042602
		2024.8257	0.049318
	High mass (> 10 kDa)	97208.3575	0.009639
		94065.66	0.038191
		94533.3665	0.046211
